# Advances in Research on Human Meningiomas

**DOI:** 10.3390/cancers12092702

**Published:** 2020-09-21

**Authors:** Sverre Helge Torp, David Scheie

**Affiliations:** 1 Department of Clinical and Molecular Medicine, Faculty of Medicine and Health Sciences, Norwegian University of Science and Technology (NTNU), 7491 Trondheim, Norway; 2 Department of Pathology, St. Olavs Hospital, Trondheim University Hospital, N-7030 Trondheim, Norway; 3 Department of Pathology, Righospitalet, Copenhagen University Hospital, Frederik Vs vej 11, 2100 Copenhagen, Denmark; david.scheie@regionh.dk

Meningiomas are the most common intracranial tumours in humans, constituting more than one third. They occur primarily in adults, more often in females, with a peak incidence in the sixth decade. Most meningiomas are solitary and well circumscribed, they are slow growing and compress and displace the surrounding brain tissue rather than infiltrate. Brain infiltration is, however, associated with a more aggressive phenotype with increased risk of recurrence. On the other hand, meningiomas may infiltrate the dura, venous sinuses, adjacent soft tissue, nerves and arteries without necessarily being associated with aggressive tumour biology, though at the same time, preventing complete resection with potential for tumour relapse.

The cellular origin of human meningiomas is thought to be the arachnoid cap cells as they share several morphologic, immunohistochemical and ultrastructural features. Furthermore, since these arachnoid cells display a wide range of epithelial-, mesenchymal- and monocyte-like properties, meningiomas may display a variegated histology with several subtypes, providing problematic differential diagnoses.

Until recently, classification of brain tumours has been based on histopathological criteria that rely heavily on the tissue of origin. However, in the latest (2016) World Health Organization (WHO) classification, molecular parameters were introduced to define several tumour entities. Meningiomas did not undergo revision, apart from reclassification of brain infiltrative meningiomas, that were seen to be aggressive and thus considered as atypical, WHO grade II tumours. Accordingly, meningiomas are classified and graded based solely on subjectively assessed histopathological features, giving considerable interobserver and sampling bias. Despite this, there is a significant association between histology and outcome in human meningiomas, resulting in the WHO classification scheme that stratifies them into three major groups, depicted as WHO grade I (benign), II (atypical (intermediate)) and III (malignant/anaplastic). However, given that about 20% of meningiomas with a benign histology and up to about 50% of atypical grade II meningiomas recur, the current WHO 2016 classification system for meningiomas is not optimal. In order to improve prognostication and diagnostics of these tumours, novel biomarkers are required. Various groups of biomarkers have been proposed, such as proliferation markers, notably Ki-67/MIB-1. However, these biomarkers have been shown to be encumbered with interlaboratory variation and considerable overlap between malignancy groups. Similarly, growth factor receptors have also been examined without demonstrable clinical significance. Concerning molecular genetics, losses of 22q and *NF2* gene mutations are the most common genetic alterations in meningiomas, and with increasing aggressiveness and tumour grade, the number of abnormal genetic alterations increases. Yet, apart from mutations in *BAP1*, *SMARC1* and *TERTp*, few molecular genetic events have been significantly associated with a more aggressive tumour biology. Recently, DNA methylation profiling has been shown to more accurately predict tumour recurrence and prognosis compared with WHO classification.

Based on factors such as tumour size, location and histological grade, some meningiomas can result in considerable patient morbidity and mortality. Gross total surgical resection is the primary treatment. Varying rates of recurrence and progression may require repeated surgery and even radiotherapy, but all these treatment modalities are associated with various complications and adverse events. Actually, data for the best follow-up and treatment are rather weak. Related to the wide range of clinical behaviour among WHO grade I and II meningiomas, there is an urgent need to discover novel protein biomarkers and mutations that can better stratify meningioma patients for optimal surgical intervention and follow-up. Thus, one may hope that this Special Issue will shed light on various aspects of meningioma biology in our effort to improve the clinical management of these tumours.

